# Effect of Programed Walking Exercise Using Bot Fit in Younger Adults

**DOI:** 10.1186/s40798-024-00773-x

**Published:** 2024-10-14

**Authors:** Su-Hyun Lee, Eunmi Kim, Jinuk Kim, Dongwoo Kim, Dokwan Lee, Hwang-Jae Lee, Yun-Hee Kim

**Affiliations:** 1 Department of Physical and Rehabilitation Medicine, Sungkyunkwan University School of Medicine, Suwon, 16419 Republic of Korea; 2 Center for Neuroscience Imaging Research, Institute for Basic Science, Suwon, 16419 Republic of Korea; 3 Bot Fit T/F, New Biz T/F, Samsung Electronics, Suwon, 16677 Republic of Korea; 4 Myongii Choonhey Rehabilitation Hospital, Seoul, 07378 Republic of Korea

**Keywords:** Aerobic, Resistance training, Exoskeleton device, Muscle strength, Pelvic

## Abstract

**Introduction:**

Physical inactivity and sedentary behavior both increase the risk of chronic disease and mortality. Regular participation in physical activity and reducing sedentary behavior play important roles in maintaining physical health and disease prevention.

**Objective:**

The purpose of this study was to investigate the effect of programed walking exercise using a wearable hip exoskeleton, Bot Fit on muscle strength, muscle effort, and the kinematics of the pelvis in younger adults.

**Methods:**

We designed three parallel experimental conditions and randomly assigned participants to one of three groups: those assigned to exercise using an interval program of Bot Fit (interval group), those who used a power program of Bot Fit (power group), and a control group who exercised without Bot Fit. A total of 45 young adults participated in 18 exercise-intervention sessions over six weeks, and all participants were assessed at two time points: before and after the 18 exercise sessions. Each assessment evaluated muscle strength, muscle effort, and the kinematics of the pelvis during walking. In addition, the number of steps, distance, energy expenditure, and heart rate for 30 min during the exercise sessions were recorded.

**Results:**

A significant increase in the maximum voluntary contraction (MVC) of the left biceps femoris (BF) was evident in the interval group, while significant changes in the MVC of the bilateral BF were seen in the power group after Bot Fit exercise. A significant decrease of muscle effort in the right BF in the interval group and right lumbar erector spinae and bilateral BF in the power group were also observed. In addition, the symmetry index of pelvic tilt significantly improved in the interval group, and greater exercise volume and intensity in both the interval and power groups compared with the control group were confirmed as measured by the number of steps, distance, energy expenditure, and heart rate.

**Conclusions:**

The results of this study confirmed the beneficial effect of programed walking exercise using the Bot Fit on muscle strength of trunk and lower extremities, muscle effort, and pelvic movement symmetry in younger adults. Personalized exercise programs can be provided for younger adults using various resistance or assistance modes of robotic device with the Bot Fit.

**Trial Registration:**

ClinicalTrials.gov, NCT05862077. Registered 22 March 2022, https://register.clinicaltrials.gov/.

## Key Points

• Bot Fit exercise offers several key advantages over exercise without a Bot Fit in terms of muscle strength, muscle effort, and pelvic symmetry during walking in younger adults.
• Greater exercise volume and intensity in interval and power groups who exercised with Bot Fit were confirmed through number of steps, distance, energy expenditure, and heart rate compared to control group who exercised without Bot Fit.

## Introduction

Low levels of physical activity and sedentary behavior (time spent sitting, as distinct from lack of physical activity) both increase the risk of chronic disease and mortality [1]. Worldwide, one in five adults are physically inactive, and sedentary lifestyles are becoming more frequent [2]. Recent updates to physical activity guidelines highlight the importance of reducing sedentary time [3]. Regular participation in physical activity and reducing sedentary behavior play important roles in physical health and disease prevention [4]. Current global recommendations state that adults aged 18–64 years should engage in at least 150 min of moderate-intensity aerobic physical activity, or at least 75 min of vigorous-intensity aerobic physical activity, or an equivalent combination of both a week, as well as muscle-strengthening activities involving major muscle groups two or more days a week [5]. Although many health organizations, including the World Health Organization (WHO), emphasize the importance of physical activity, the number of individuals who currently do not meet the recommendations for physical activity continues to grow worldwide. Recent global estimates show that one in four (27.5%) adults [6] and more than three-quarters (81%) of adolescents [7] do not meet the aerobic exercise provisions of the 2010 Global Recommendations on Physical Activity for Health [8].

Research suggests that regular aerobic exercise can prevent weight gain and improve physical fitness, cardiorespiratory health, executive functioning, and brain health even in healthy populations [9–12]. Additionally, strong clinical evidence and emerging epidemiological data show that muscle-strengthening exercise is independently associated with multiple health outcomes, including reduced mortality risk and incidence of diabetes, as well as enhanced cardiometabolic, musculoskeletal, and mental health [13–17]. However, muscle-strengthening exercise has been overlooked in public health approaches to chronic-disease prevention despite numerous independent health benefits compared with the attention devoted to aerobic physical activity. Moreover, recent health surveillance data from several countries show that only 10–30% of adults meet the guidelines for muscle-strengthening exercise, a far lower proportion than those meeting aerobic physical activity guidelines (approximately 50%) [18, 19].

Physical activity recommendations in public health campaigns have focused on promoting moderate-to-vigorous-intensity exercise since the 1970s. However, over the past decade, the value of muscle-strengthening exercise has been recognized, with the combination of muscle-strengthening exercise and aerobic physical activity being a recent addition to physical activity guidelines [20]. Recent epidemiological studies suggested that, compared with engaging in either moderate-to-vigorous-intensity aerobic physical activity or muscle-strengthening exercise alone, a combination of both has more favorable effects on cardiometabolic biomarkers, lean muscle mass, and mental health [21–23]. Moreover, concurrent exercise, an integrative exercise modality that combines aerobic physical activity with muscle-strengthening exercise, is a time-efficient strategy that fits into modern busy lifestyles and provide the benefits of both interventions [24, 25].

Resistance training during aerobic physical activity, such as walking with weights, is a well-recognized method of increasing metabolic cost while improving muscle strength, overground walking performance, and endurance in healthy as well as neurologically impaired individuals [26–29]. A few studies have used weights attached to a subject’s waist and connected to a pulley system to examine the efficacy of resistance training while walking on a treadmill [27, 28, 30]. Other studies have added weights to subjects’ limb segments as they walk [31] or utilised walking in water tanks [32]. However, conventional training is often performed using additional loads such as resistance bands, weighted cuffs, or a backpack on the body, all of which tend to be bulky and less stable (i.e., large resistive forces are only possible with excessively large weights). Moreover, these methods typically vary task intensity through progressive schemes, such as increasing band stiffness or adding more weight over time [26, 31, 33].

Wearable robotic systems, particularly those that provide resistance targeting specific muscle groups and functions, have emerged as potential tools to more effectively target the appropriate timing of the activity of specific muscle groups in a functional context [34]. In this study, we used a wearable robotic hip exoskeleton, Bot Fit (Samsung Electronics Co., Ltd., Suwon, Republic of Korea), which can produce assistive and resistive torque during walking, to investigate the effects of Bot Fit exercise programs. In our previous studies, we demonstrated the effects of assistance and resistance algorithm of Bot Fit [35–37]. In essence, the assistance algorithm of the Bot Fit reduced respiratory metabolic energy consumption while walking at a comfortable speed, improved gait function (gait speed, stride length, and gait symmetry) and pelvic mobility, and resulted in better gait posture and more efficient movement. The resistance algorithm simultaneously stimulated muscle activation in the lower extremities and trunk during walking and provided greater strengthening benefits in the lower extremity muscles than conventional walking exercises. In this study, we designed two unique Bot Fit exercise programs, the interval and power programs, by specifically combining the assistance and resistance algorithms. The purpose of this study was to investigate the effects of Bot Fit exercise programs on the muscle strength, muscle effort, and kinematics of the pelvis during walking in younger adults. The findings will help in the development of a personalized exercise program using Bot Fit.

## Methods

### Study Design and Participants

This study used a single-blinded (evaluator), randomized, controlled, three-group parallel design. Based on medical history and functional assessments, we excluded individuals with a history of neurological and musculoskeletal disorders that affect walking capacity, efficiency, or endurance. Eligible subjects were healthy and between 19 and 65 years of age without any history of central nervous system disease [36, 38]. The participations were randomly assigned to one of three groups: those assigned to exercise using a Bot Fit interval program (interval group, *n* = 15), those who used a Bot Fit power program (power group, *n* = 15), and a control group (*n* = 15) that exercised without Bot Fit using a computer-generated 1:1:1 allocation. The characteristics of the 45 participants are shown in Table 1. Study procedures were approved by the ethics committee of the Sungkyunkwan University Institutional Review Board (approval number: 2023-03-005) and registered with ClinicalTrials.gov (NCT05862077). Written informed consent was obtained from all participants before they entered the study, and all methods were carried out in accordance with the approved study protocol.

**Table 1:** Baseline characteristics of participants (*N* = 45)

Characteristics	Control	Interval	Power	χ^2^/F	*P*
Age, years	38.47 (4.82)	36.07 (2.46)	37.40 (5.79)	1.035	0.364
Sex (male/female)	14/1	10/5	11/4	3.343	0.188
Height, cm	174.30 (6.94)	172.03 (8.80)	171.53 (9.10)	0.469	0.629
Weight, kg	77.41 (11.13)	70.99 (13.99)	76.53 (13.09)	1.111	0.339
Body mass index, kg/m^2^	25.41 (2.76)	23.78 (3.12)	25.87 (2.94)	2.093	0.136

Data are expressed as mean (standard deviation)

### Exercise Intervention

All subjects in the interval and power groups performed community-mobility routines (walking in the Samsung Digital City, including level walking, slope and stair walking, and crossing the cross walk) for 30 min with a Bot Fit while the control group did the same without a Bot Fit. The interval group exercised using the interval exercise program, which repeats rapid walking in resistance mode and slow walking in assistance mode, while the power group exercised using the power exercise program, which repeats rapid walking in resistance mode and rapid walking in assistance mode. The interval and power exercise programs consist of a total of four sessions, with each session including a resistance mode for 4 min and 30 s and an assistance mode for 3 min. During the two Bot Fit exercise programs, walking speed is controlled by heart rate (HR) measured by a watch-type HR sensor (Samsung Galaxy Watch 4, Samsung Electronics Co., Ltd., Suwon, Republic of Korea) that communicates with Bot Fit. In slow walking, the speed is adjusted to maintain an average HR in the 70–80% range of HRmax, and in fast walking, the speed is adjusted to maintain an average HR in the 80–90% range of HRmax. Subjects adjusted their own walking speed to reach target heart rate zones in each session. Bot Fit adjusts the intensity of assistive and resistive torque based on HR data measured by the HR sensor to help subjects reach their target heart rate zones.

### Bot Fit Wearable Hip-assist Robot

**Fig. 1:**
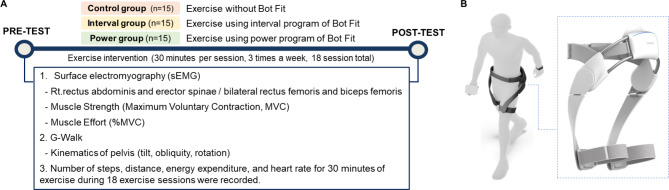
(**A**) Experimental protocol; (**B**) Bot Fit wearable hip exoskeleton

The Bot Fit is a wearable hip-type robot designed to promote health in younger adults. The device is fastened around the wearer’s waist and thighs to assist or resist hip-joint flexion and extension. The assistive and resistive torque is transmitted to the user’s thighs through an exoskeletal frame (Fig. 1). Users can operate the device and change the exercise program settings using an application on a mobile phone and smart watch.

Assistive and resistive hip torque are generated by Bot Fit through a specialized control algorithm called Delayed Output Feedback Control (DOFC), which is a time-delayed, self-feedback controller designed specifically for walking with wearable robots [39]. Briefly, the angles of the right and left hips are obtained using embedded angular position sensors. Then, based on the measured angle between the bilateral hip joint in real time, assistive and resistive torque are generated by combining appropriate time delays for torque adaptation timing with either positive or negative feedback gains to modulate torque level. The maximum torque generated by DOFC algorithm is 6 N/m.

### Assessment Tools and Data Collection

Participants were evaluated at two time points: before (pre) and after (post) 18 exercise-intervention sessions. Each assessment evaluated muscle strength, muscle effort, and the kinematics of the pelvis during walking. In addition, the number of steps, distance, energy expenditure, and HR for each 30-min exercise session were recorded.

To measure surface electromyography (sEMG) signals (Noraxon USA Inc., Scottsdale, AZ, USA), bipolar surface electrodes (Ag/AgCl) were positioned on the right rectus abdominis (RA) and lumbar erector spinae (LES) and the bilateral rectus femoris (RF) and biceps femoris (BF) of subjects following guidance by the Surface ElectroMyoGraphy for Non-Invasive Assessment of Muscles (SENIAM) project for reliable sensor placement [40]. In addition, footswitches (Model 500 DTS FootSwitch; Noraxon USA Inc.) were placed on the bilateral toe and heel to record the timing of the stance and swing gait phases while walking. Before placing sEMG sensors, subjects’ skin was shaved, abraded, and cleaned with alcohol to reduce surface impedance. In this study, muscle strength refers to the maximum amount of force that a muscle can generate, and the value measured during maximal voluntary contraction (MVC) was used. Muscle effort refers to the relative amount of force that a muscle exerts compared to its maximum capability, and for our purposes muscle effort was defined as the percentage of MVC (% MVC) averaged across all participants. The MVC test was proposed by the SENIAM project and kinesiology guidelines and is the most widely-used normalization method [41]. To normalize the sEMG signal amplitude, three 5-s maximal muscle contraction measurement trials were performed with each muscle to determine each participant’s MVC. To reduce movement artifacts, a sampling frequency of 1,000 Hz was used, and raw sEMG signals were bandpass filtered between 10 and 350 Hz and full-wave rectified with Noraxon software (MyoResearch XP Master Edition). The root mean squared values of the signals were calculated using a sliding 100-ms window. The data were passed through a sixth-order Butterworth low-pass filter with a 6-Hz cutoff to create a linear envelope and normalized to the MVC data obtained prior to the tasks. The average normalized sEMG activity was processed within the selected phases of the gait cycle using MATLAB software (MathWorks, Inc., Natick, MA, USA) [42]. Individual gait cycles were determined using footswitch data, with each stride considered the period between successive heel strikes by the same leg. The sEMG patterns for each stride were time-normalized and expressed as a percentage of the total gait cycle (0–99%).

The three-dimensional kinematics of the pelvis (anterior-posterior tilt, up-down obliquity, and intra-extra rotation) and pelvic symmetry index (SI) were measured using a wireless G-Walk wearable sensor (BTS Bioengineering S.p.A., Milan, Italy), which consists of a triaxial accelerometer, a magnetic sensor, and a triaxial gyroscope. The G-Walk sensor was placed on the subject’s waist using a semi-elastic belt covering the L4-L5 intervertebral space to acquire the acceleration values for the three anatomical axes (antero-posterior, medio-lateral and vertebral). The collected data, transmitted to a personal computer via Bluetooth and processed with dedicated BTS G-Walk software, allowed us to obtain a set of gait parameters from which the kinematics of the pelvis (degree of tilt, obliquity, and rotation) and SI (%) could be analyzed. SI quantifies the similarity of the profiles of the right and left curves. If the two curves overlap perfectly, the index score is 100, meaning that the two curves have the same value frame by frame. The pelvis position in three planes comprises the pelvic tilt in the sagittal plain, pelvic obliquity in the coronal plane, and pelvic rotation in the transverse plane. For pelvic tilt, SI values higher than 40 can be considered normal. For the pelvic obliquity and rotation angles, a normal SI value is 80–100 (according to guidelines supplied by the manufacturer, BTS Bioengineering S.p.A.). The BTS G-Walk device uses a mathematical correlation function applied to the two curves: symmetric index = (corr + 1) × 100/2, where corr is the Pearson correlation coefficient between the mean left and right normalized anteroposterior acceleration signals (according to the manufacturer’s guidelines). Subjects were asked to walk 10 m in one direction and then turn around and walk 10 m back at a self-selected speed, as naturally as possible; muscle effort and pelvic kinematics were measured simultaneously.

The number of steps, distance, energy expenditure, and average HR during 30 min of exercise were measured using a Samsung Galaxy Watch 4 linked to a mobile phone Bot Fit application.

### Statistical Analyses

Statistical analyses were performed using SPSS version 22.0 (IBM, Armonk, NY, USA), with a significance level of 0.05. Descriptive statistics are expressed as means and standard deviation. After examining the distribution of the data for normality, we chose parametric tests for MVC and pelvic movement and non-parametric tests for muscle effort (% MVC). One-way analysis of variance (ANOVA) for continuous variables and chi-square tests for categorical variables were used to compare participants’ baseline characteristics. To compare the outcome measures before and after exercise in each group, paired t-tests and Wilcoxon signed-rank tests were used. One-way ANOVA and Kruskal–Wallis tests were used to determine statistically significant differences among groups. In addition, repeated-measures ANOVA was used to examine the main effects of exercise over time, including groups and time points. Post hoc tests were used to identify differences among the group means, and the significance levels of the tests were adjusted using Bonferroni correction.

## Results

### Characteristics of the Participants

In the present study, 45 participants were enrolled, with 15 individuals assigned to each group. One-way ANOVA and Chi-square analysis revealed no significant differences among the three groups in terms of age, sex, height, weight, and BMI (Table 1).

### Effects of Bot Fit Exercise on Muscle Strength and Muscle Effort

**Fig. 2:**
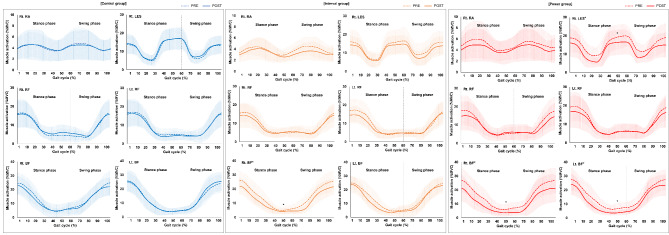
Effects of Bot Fit on muscle effort (% MVC). Muscle effort during the whole gait cycle (100%) decreased significantly in the right BF in the interval group and in the right LES and bilateral BF in the power group after Bot Fit exercise. PRE: before the 1st exercise intervention session, POST: after the 18th exercise intervention session. ^*^Significant change compared with pre (*P* < 0.05, Wilcoxon signed-rank tests). MVC: maximum voluntary contraction, Rt: right, Lt: left, RA: rectus abdominis, LES: lumbar extensor spinae, RF: rectus femoris, BF: biceps femoris

Table 2; Fig. 2 present the specific values for MVC (µV) and muscle effort (% MVC) at the respective pre– and post–time points. Significant improvements in the MVC of the left BF were seen in the interval group (*P* = 0.017), while the power group showed significant changes in the MVC of the bilateral BF after Bot Fit exercise (*P* = 0.045 on the right BF and *P* = 0.049 on the left BF). However, no significant changes were evident in the control group. Muscle effort during the total gait cycle (100%) decreased significantly in the right BF in the interval group (*P* = 0.033) and in the right LES (*P* = 0.001) and bilateral BF (*P* = 0.002 on the right BF and *P* = 0.002 on the left BF) in the power group after Bot Fit exercise. In contrast, the control group showed no significant muscle effort decreases. Significant group × time interactions were also found in muscle effort in the right LES (*P* = 0.003) and bilateral BF (*P* = 0.021 on the right BF and *P* = 0.005 on the left BF), and the interval and power groups experienced greater changes compared with the control group.

**Table 2:** Effects of Bot Fit exercise on muscle strength (*N* = 45)

MVC, µV	Control		Interval		Power			Between-group	
	Pre	Post	Pre	Post	Pre		Post	F	*P*
Rt. RA	153.40 (82.69)	149.47 (76.70)	134.01 (63.46)	136.37 (75.17)	118.49 (46.03)	128.37 (53.81)		0.326	0.724
Rt. LES	190.11 (111.68)	184.69 (123.74)	161.51 (80.59)	161.62 (76.25)	151.45 (76.69)	164.23 (73.79)		0.364	0.697
Rt. RF	214.05 (79.65)	216.85 (75.37)	168.62 (57.10)	176.39 (70.18)	217.10 (80.57)	232.36 (87.35)		0.176	0.840
Lt. RF	197.05 (78.26)	198.64 (63.98)	191.64 (63.65)	199.08 (91.27)	204.77 (78.67)	217.25 (94.99)		0.108	0.898
Rt. BF	240.96 (112.56)	251.35 (124.90)	205.09 (79.35)	245.28 (126.31)	209.25 (99.41)	250.44 (102.92)^*^		0.776	0.468
Lt. BF	223.40 (94.62)	242.97 (114.18)	195.47 (91.37)	236.00 (96.07)^*^	183.29 (75.20)	225.50 (87.63)^*^		0.728	0.490

Data are expressed as mean (standard deviation)

^*^Significant change compared with Pre (*P* < 0.05, paired t-tests)

MVC: maximum voluntary contraction, Rt: right, Lt: left, RA: rectus abdominis, LES: lumbar extensor spinae, RF: rectus femoris, BF: biceps femoris

### Effects of Bot Fit Exercise on Kinematics of the Pelvis

**Fig. 3:**
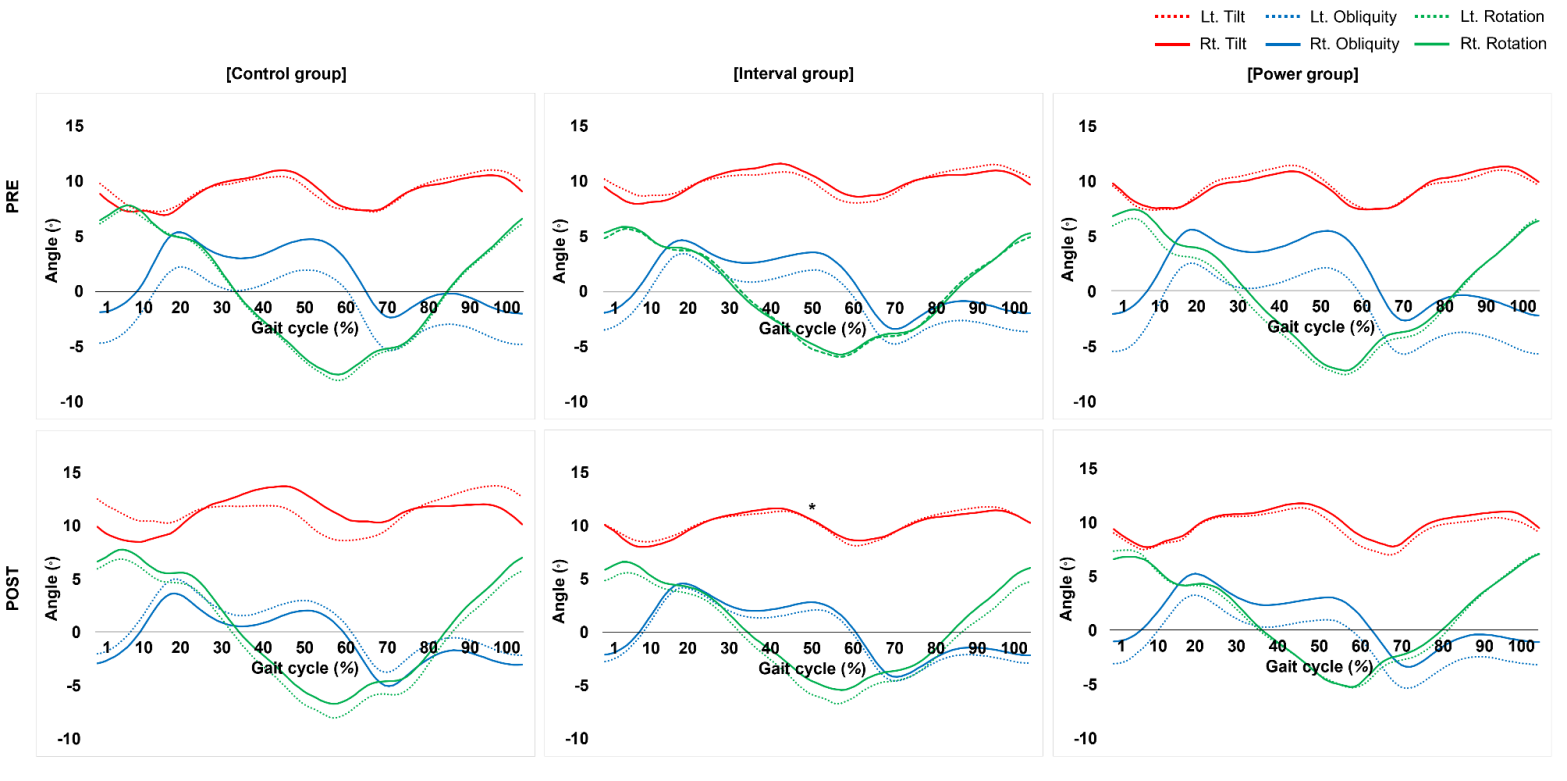
Kinematics of pelvis during walking. The symmetry index of pelvic tilt increased significantly in post- compared to pre-intervention time points in the interval group. PRE: before the 1st exercise intervention session, POST: after the 18th exercise intervention session. ^*^Significant change compared with pre (*P* < 0.05, paired t-tests). Rt: right, Lt: left

Specific values for pelvic SI and kinematics of the pelvis are presented in Table 3; Fig. 3. The SI of pelvic tilt tended to increase in post compared to pre time points in the interval and power groups (by 23.80% and 23.04%, respectively), but the increase was significantly different only in the interval group (*P* = 0.046).

**Table 3:** Effects of Bot Fit exercise on pelvic symmetry index (*N* = 45)

	Control		Interval		Power		Between-group	
	Pre	Post	Pre	Post	Pre	Post	F	*P*
Tilt	60.77 (17.17)	54.53 (20.23)	53.73 (28.68)	71.89 (21.07)^*^	60.19 (27.56)	74.06 (15.48)	2.659	0.082
Obliquity	97.41 (1.80)	98.02 (0.83)	98.37 (0.96)	98.46 (1.05)	98.39 (0.79)	98.63 (0.98)	0.364	0.697
Rotation	98.41 (1.09)	98.65 (0.88)	98.52 (0.59)	98.49 (0.93)	96.57 (6.85)	98.66 (0.79)	1.235	0.301

Data are expressed as mean (standard deviation)

^*^Significant change compared with Pre (*P* < 0.05, paired t-tests)

### Number of Steps, Distance, Energy Expenditure, and Heart Rate during Exercise

**Fig. 4:**
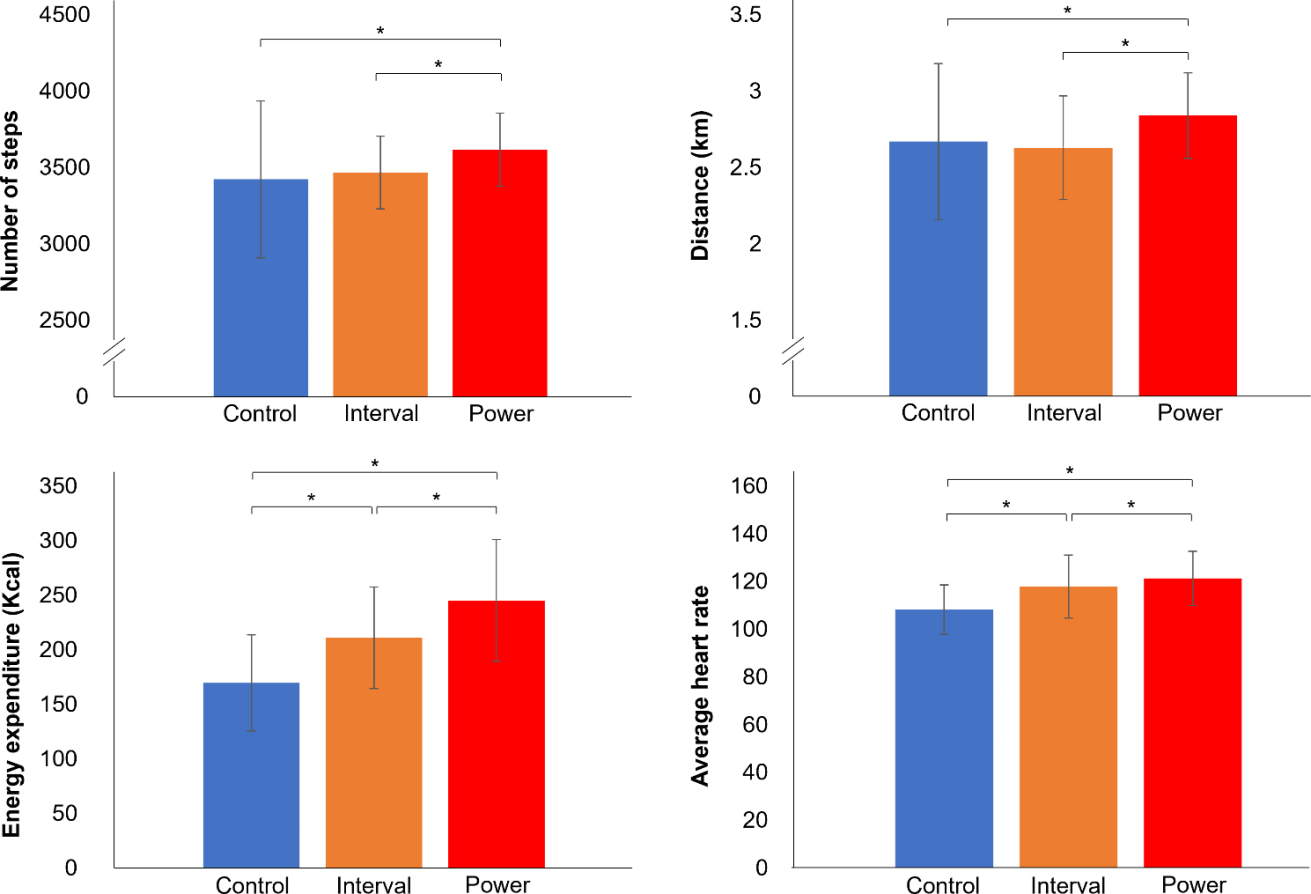
Number of steps, distance, energy expenditure, and heart rate during exercise. The number of steps and distance were significantly higher in the power group than in the control and interval groups. Energy expenditure and average heart rate were significantly higher in the power group than in the interval and control groups, and the energy expenditure and average heart rate of the interval group were also significantly higher than those of the control group. ^*^Significant change compared with the control group or interval group (*P* < 0.05)

Figure 4 presents values for the number of steps, distance, energy expenditure, and average HR during exercise in each group. The number of steps and distance were significantly higher in the power group than in the control and interval groups (*P* < 0.001). The number of steps was 5.66% greater compared with the control group and 4.33% greater than the interval group, and the distance was 6.37% longer compared with the control group and 7.98% longer than the interval group. Energy expenditure was significantly higher in the power group than in the interval and control groups, and energy expenditure of the interval group was also significantly higher than that of the control group (*P* < 0.001): by 24.30% in the interval group and 44.38% in the power group compared with the control group. The maximum heart rate (HR_max_) averages predicted by the [207 − 0.7 × age] equation [43] were 180.1 in the control group, 181.8 in the interval group, and 180.8 in the power group. The ranges in moderate exercise intensity (64–76% of HR_max_) were from 115.3 to 136.9 in the control group, 116.3 to 138.1 in the interval group, and 115.7 to 137.4 in the power group. The average HR during exercise was significantly higher in the power group than in the interval (*P* = 0.023) and control groups (*P* < 0.001), and it was also significantly higher in the interval group than in the control group (*P* < 0.001): by 8.99% in the interval and 12.07% in the power group compared with the control group. In addition, the average HRs in the interval group (117.99 ± 13.20) and power group (121.33 ± 11.33) were in the range of moderate exercise intensity, but the average HR in the control group (108.26 ± 10.31) was not in the range of moderate exercise intensity.

## Discussion

This study investigated the effects of robotic-resisted exercise with a novel wearable hip exoskeleton, Bot Fit, on muscle strength, muscle effort, and the kinematics of the pelvis during walking in younger adults. The results of this study suggest that walking using a Bot Fit exercise programs offers several key advantages over exercise without a Bot Fit in terms of muscle strength, muscle effort, and pelvic symmetry during walking in younger adults. The interval group experienced a significant increase in the MVC of the left BF and the power group showed significant changes in the MVC of the bilateral BF after Bot Fit exercise. Significant decreases in muscle effort in the right BF in the interval group and the right LES and bilateral BF in the power group were observed. In addition, the SI of pelvic tilt improved significantly in the interval group, and greater exercise volume and intensity in the interval and power groups were confirmed through the number of steps, distance, energy expenditure, and HR.

The benefits of regular aerobic moderate-to-vigorous physical activity are well established and embraced by the Physical Activity Guidelines for Americans and the WHO 2020 Global Recommendations on Physical Activity for Health [44, 45]. The evidence that activities such as brisk walking, cycling, sports, and planned exercise reduce the risk of cardiovascular disease, stroke, type 2 diabetes, some cancers, and all-cause mortality has accumulated since the 1950s [44]. Recommendations for aerobic physical activity have been strengthened and refined since the 1970s, but the recommendation for adults to engage in muscle-strengthening activities was first included only in the 2008 Physical Activity Guidelines for Americans [46]. Unlike the decades of epidemiological research on moderate-to-vigorous aerobic activity, comparable research on muscle-strengthening exercise is limited. However, the role of muscular strength in the prevention of chronic disease in adults has been increasingly recognized [47], and the inclusion of resistance training in exercise programs that promote adult health has been endorsed by several organizations [48]. In this study, the interval and power groups simultaneously performed aerobic physical activity (walking) and muscle-strengthening exercise with Bot Fit, while the control group performed only walking without a Bot Fit. The MVC of the left BF in the interval group and the MVC of the bilateral BF in the power group increased significantly after Bot Fit exercise, but there was no change in MVC after exercise in the control group. In addition, significant decreases in muscle effort in the right BF in the interval group and the right LES and bilateral BF in the power group were observed, but there was no change in muscle effort after exercise in the control group. These results imply that the assistance and resistance imposed by a Bot Fit, which generates hip-joint flexion and extension-assistive and resistive torque during walking, affected the MVC of the hip extension muscle, enhancing muscle strength and decreasing effort of BF. Concurrent aerobic and resistance exercise has been shown to rapidly increase muscular strength and is essential for improving sports performance, injury prevention and rehabilitation, and health in all populations [49, 50]. It is well established that aerobic and resistance exercise leads to changes in the nervous system, which plays an important role in the development of strength [51, 52]. The increased MVC caused through Bot Fit exercise is expected to promote muscle strength in the BF by improving neural adaptations such as motor unit activation and synchronization, and by decreasing presynaptic inhibition [53]. Increases in muscle strength reduce relative muscle effort, which leads to improved efficiency and endurance, and a reduced likelihood of fatigue and injury [54, 55]. Our results indicate that Bot Fit exercise can increase muscle strength and help individuals walk more efficiently by reducing the muscle effort required.

Control of pelvis alignment is necessary for efficient movement and walking, and if it is not properly controlled during walking, the speed, stability, and efficiency of the walk decreases [56–58]. Asymmetry of the pelvis can be associated with the development of non-specific chronic low back pain, caused by incorrect mechanical load on the body, which increases the stress on soft tissues in the lumbar Sects. [59, 60]. The increased use of sitting positions in daily life and sedentary behavior among young adults tends to increase the asymmetric load exerted on the spine and disrupt pelvic symmetry [61, 62]. In this study, the SI of pelvic tilt increased in the interval and power groups (by 23.80% and by 23.04%, respectively) after Bot Fit exercise, but the SI of pelvic tilt decreased in the control group (by 10.27%). The increase in the SI of pelvic tilt in the two groups that exercised using a Bot Fit can be attributed to the strengthened abdominal and hip muscles involved in pelvic tilt due to aerobic and resistance exercise using a Bot Fit. In addition, a Bot Fit can stimulate and train a wearer’s muscles by providing controllable assistive and resistive force and torque at the hip joint. These sensory inputs provide continuous feedback to correct movements and improve proprioception, and may influence pelvic symmetry during walking.

The most commonly used metric for exercise intensity is HR, which is easy to monitor and stable during exercise [63]. It is generally accepted that the relationship between HR/workload and oxygen consumption (and therefore caloric expenditure) is linear, and HR therefore accurately reflects workload and exercise intensity. As such, it is often used to prescribe exercise and track changes in the training status of athletes [64, 65]. In this study, the exercise volume and intensity of each group were measured using the number of steps, distance, energy expenditure, and HR. The number of steps and distance during exercise were significantly higher in the power group than in the interval and control groups, and the average HR and energy expenditure during exercise were significantly higher in the interval and power groups compared with the control group. A Bot Fit can provide extensive, repetitive, task-specific walking exercise that can increase exercise intensity, which in turn affects HR and energy expenditure during exercise. In addition, the Bot Fit exercise programs increase exercise volume by providing resistance and encouraging walking at a speed above a certain level. Use of the Bot Fit during exercise can enhance the physiologic effects of exercise compared with the same exercise performed without a Bot Fit.

This study has limitations related to inadequate control of everyday activity levels of the participants beyond the exercise-intervention session. During the long-term intervention period, other factors that may have affected the study outcome, such as an individual’s overall amount of physical activity, were not controlled or monitored. Second, the statistical power of this study was low because of the small number of participants, and our results cannot be generalized to the entire young population. Nevertheless, this study demonstrates that use of a Bot Fit while walking can improve muscle strength and effort as well as the symmetry of pelvic movement compared with the same exercise without a Bot Fit. Further research could consider a cross-over design to increase the number of participants in each exercise program and increase the statistical power of the analysis.

## Conclusions

This study was a randomized controlled trial evaluating the effects of robotic-resisted exercise with the Bot Fit in younger adults. The results of this study confirmed the beneficial effect of the Bot Fit on muscle strength, walking efficiency, and pelvic movement symmetry in younger adults. Personalized exercise programs using different exercise protocols with the Bot Fit can therefore improve physical performance and gait symmetry in younger adults.

## Data Availability

The datasets used and/or analyzed during the current study are available from the corresponding author on reasonable request.

## Abbreviations

sEMG: Surface electromyography
RA: Rectus abdominis
LES: Lumbar erector spinae
RF: Rectus femoris
BF: Biceps femoris
MVC: Maximum voluntary contraction
SI: Symmetry index
HR: Heart rate
